# The malignant potential of HIV-associated Kaposi sarcoma

**DOI:** 10.1186/1475-2867-8-14

**Published:** 2008-10-31

**Authors:** Neil H Wood, Liviu Feller

**Affiliations:** 1 Department of Periodontology and Oral Medicine, School of Dentistry, University of Limpopo (Medunsa Campus) Pretoria, South Africa

## Abstract

Human herpesvirus (HHV)-8 associated oncogenesis, a state of immune impairment, a local inflammatory environment, angiogenesis and HIV infection occurring concurrently are important factors for the development of HIV-associated Kaposi sarcoma (KS).

Activation of the interleukin (IL)-6 receptor signalling pathway and constitutive signalling of viral G protein-coupled receptor (vGPCR) play an important role in the activation, proliferation and transformation of HHV-8 infected endothelial cells thus contributing to the initiation and progression of KS. HIV-tat protein, HIV-induced immune suppression and a hyperinflammatory state facilitate the oncogenic activity of HHV-8.

In this article we reviewed some aspects of HIV-KS pathogenesis and tried to establish, according to the available information in the literature, whether HIV-KS is a monoclonal neoplasm or a benign angioproliferative disorder.

From the data of this review it is evident that most of the HIV-KS lesions are oligoclonal in origin. It remains to be demonstrated whether these multiple monoclonal populations of cells are neoplastic, harbouring specific cytogenetic alterations such as mutations, rearrangements and amplifications, or are, as the current evidence shows, the result of HHV-8 induced intracellular signalling pathways that modulate the expression of cellular genes associated with cell cycle regulation, apoptosis, inflammatory response and angiogenesis, and represent a reactive angioproliferative disorder.

## Background

The clinical course of human immunodeficiency virus (HIV)-associated Kaposi sarcoma (HIV-KS) ranges in severity from a mild, slowly progressing to a rapidly progressing life threatening condition. Invariably, without treatment, the overall prognosis of HIV-KS is poor [1].

The clonal nature of KS is controversial and the exact cell of origin of KS is not clear. The cell of origin may arise from lymphatic or blood endothelium, vascular smooth muscle cells, mesenchymal cells or a combination of these cells [2]. However, the current opinion is that KS probably originates from lymphatic endothelium [3,4]. HHV-8 infection of blood vascular endothelial cells leads to lymphatic endothelial reprogramming of these cells [3,4], and it is suggested that HHV-8 may preferentially infect endothelial cell precursors and mediate their differentiation towards a lymphatic endothelial cell genotype [4,5].

HHV-8 induced inflammation, angiogenesis and oncogenesis are critical for the development of HIV-KS [6]. HHV-8 expresses several oncogenic proteins that are homologous to cellular proto-oncogenes. These include vIL-6, vIL-8 receptor, chemokines of the macrophage inflammatory protein (MIP) family, cell-cycle regulators of the cyclin family and anti-apoptotic proteins of the *bcl-2 *family. These biological mediators have the capacity to activate several intracellular signaling pathways that may bring about a hyperinflammatory state, angiogenesis and transformation of endothelial cells. These events are central to the initiation and promotion of HIV-KS [1].

It is unclear whether HIV-KS is a true neoplastic entity arising from a clonal expansion of a single HHV-8 infected cell, or is a benign angioproliferative disorder. The demonstration that HIV-KS lesions are monoclonal will provide further evidence on the probable malignant nature of HIV-KS, since monoclonality is an important feature of cancer.

In this article we reviewed the available data in the literature regarding the clonal nature of HIV-KS.

It is probable that HIV-KS starts as a polyclonal inflammatory hyperplasia, and a subset of advanced HIV-KS lesional cells undergo malignant transformation, which may explain the aggressive clinical course of such cases [7].

## HHV-8

HHV-8 is aetiologically linked to KS, but is not sufficient on its own to transform HHV-8 infected endothelial cells and to initiate the development of KS. Other factors including HIV-tat protein, a state of immune suppression and dysregulation of cytokines, growth factors and adhesion molecule functions cooperate with HHV-8 to drive the initiation and promotion of HIV-KS [8].

HHV-8 gene expression is tightly controlled and occurs in two phases: the latent phase that facilitates viral genome persistence, and the lytic phase which consists of viral replication, host cell destruction and viral release [9]. During the early stages of HHV-8 infection, cellular gene expression dominates, but as the viral infection progresses, and viral DNA has entered the nucleus, latent and lytic viral gene expression begins to overlap with that of the host, resulting in a multifaceted intra- and intercellular signaling cascade, which in turn is controlled by protein kinases that phosphorylate serine, threonine and tyrosine residues of target cellular substrates, leading to the eventual cellular transformation [9,10].

The early KS lesion is most probably initiated by HHV-8 induced production of pro-inflammatory cytokines IL-1, IL-6, tumour necrosis factor (TNF)-α, interferon (IFN)-γ and growth factors basic fibroblast growth factor (bFGF) and vascular endothelial cell growth factor (VEGF) that causes activation of endothelial cells followed by angiogenesis (Diagram 1) [1,10-12].

In advanced KS lesions, HHV-8 oncogenes can alter the regulation of intracellular signal transduction pathways, cell cycle progression and apoptosis through the expression of several oncoproteins that are homologous to cellular proto-oncogenes, including vIL-6, vIL-8 receptor, MIP and anti-apoptotic proteins of the *bcl-2 *family. By sabotaging the regulatory mechanisms of normal cellular proliferation, differentiation and survival, the complex combined expression of host and viral oncogenes contributes to tumour development (Diagram 1) [12].

### Role of HHV-8 in the sarcomagenesis of KS

Latent and lytic HHV-8 gene products have the capacity to target cellular apoptosis and host transcription pathways. The inhibition of apoptosis leads to increased survival of the infected cell, and the hijacking of the cellular transcription machinery enables HHV-8 to overcome viral gene transcription restriction. These events promote the propagation of HHV-8 infection (Diagram 1) [9,10,13,14].

#### Latent genes

HHV-8 latent proteins, including latency associated nuclear antigen (LANA)-1, LANA-2, vCyclin, viral Fas-associated death domain-like interleukin 1 gamma-converting enzyme inhibitory protein (vFLIP) and kaposin (Table 1), are expressed in all cells of advanced HIV-KS lesions, and although their role in the sarcomagenesis of KS has been confirmed, they are necessary but not sufficient to cause the development of KS [15,16]. Tumour hyperplasia and spindle cell proliferation are induced and maintained by the action of these viral oncoproteins, either acting alone, or in combination with each other through paracrine mechanisms [15-17].

**Table 1:** HHV-8 latent genes

Genes	Products
ORF 73	LANA-1 and -2
ORF 72	vCyclin
ORF 71	vFlip
ORF K12	Kaposin

LANA-1 and LANA-2 may act as transcriptional regulators. The expression of cellular and viral genes is modified by the function of LANA [9,10]. By targeting the Rb-E2F pathway and the p53 apoptosis control pathway, LANA is able to inhibit cell death and promote cellular transformation [2,9,10]. In addition, LANA is critical for the persistence of HHV-8 episomes. By anchoring the viral episomes to the host DNA during interphase and mitosis, the efficient segregation of viral episomes to daughter cells occurs in a synchronized and non-random fashion [9].

vFLIP is able to upregulate the expression and activation of the transcription factors NF-κB and AP-1 (see lytic genes below) that play a role in tumour cell survival during the latent phase of HHV-8 infection [10,17].

Kaposin is another latent-phase gene product. This unique HHV-8 oncoprotein, encoded by open reading frame (ORF)-K12, is expressed in primary effusion lymphoma cell lines, can induce transformation in vitro, and it has been suggested that it may play a role in the development of KS [15,18,19].

#### Lytic genes

Some HHV-8 lytic genes (Table 1) play a major role in the sarcomagenesis of KS [20]. vGPCR is an HHV-8 lytic gene product that has been functionally linked to HHV-8-mediated tumorigenesis [21]. vGPCR is a member of the CXC-chemokine G protein-linked receptor family, shows significant homology to the IL-8 receptors CXCR1 and CXCR2, and exhibits ligand-independent activities. Constitutive vGPCR signaling is augmented by chemokines such as IL-8 and growth-related oncogene α that act as vGPCR agonists [15,22]. Despite the fact that the expression of vGPCR is transient and is found only in 1–6% of KS cells, vGPCR is able to initiate KS-like tumours in mice [17,23].

Through constitutive activation of intracellular signaling pathways, vGPCR has the capacity to transform endothelial cells by promoting their uncontrolled proliferation and by inhibition of apoptosis [6,11,12,15,21,22].

The increased secretion of angiogenic growth factors, chemokines and cytokines induces either enhanced proliferation of vGPCR-transformed endothelial cells in an autocrine manner, or stimulates angioproliferation of neighboring bystander cells by paracrine mechanisms (Diagram 1) [12,14-17,21,22,24-27]. In addition, vGPCR constitutive signaling may promote endothelial cell survival directly through the activation of Akt/protein kinase B pathway (Akt/PKB) (Diagram 1) [7,15,16,22].

Stimulation of vGPCR leads to the activation of NF-κB, activator protein (AP)-1 and nuclear factor of activated T cells (NFAT), which induce the endogenous expression of NF-κB and AP-1 dependant cytokines. These cytokines include IL-1, TNF-α and IL-6 as well as growth factors (Diagram 1) [17,27]. In addition, vGPCR signaling also induces the upregulation of vascular endothelial cell growth factor (VEGF) receptors. This facilitates the stimulation of endothelial cells in an autocrine and paracrine manner [20].

It is suggested that vGPCR in KS tumour cells activates Akt/PKB in a paracrine and autocrine manner to promote cell survival [16]. The autocrine pathway leads to the induction of the angiogenic cell phenotype, whereas the paracrine pathway maintains growth and proliferation through the expression and secretion of various mediators such as VEGF that promote angiogenesis (Diagram 1) [21].

The activation of the serine-threonine kinase Akt by vGPCR is critical for inhibition of apoptosis. Activated Akt/PKB brings about inhibition of different pro-apoptotic proteins, including the caspase family, and the concomitant activation of transcription factors that leads to an increase in the expression of survival genes (Diagram 1, blue arrows) [22,28].

It is suggested that through GTP binding molecules like Rac, vGPCR can activate Akt/PKB, a critical molecule in the control of cell survival and tumour development [16,22]. Small GTP-binding proteins are the links between the vGPCR and nuclear transcription factors. The activation of Rac-GTPase by vGPCR expressed on HHV-8 infected endothelial cells in KS lesions stimulates the transcription factors NF-κB and AP-1 (Diagram 1) [17]. By preventing the vGPCR activation of Rac-1, the stimulation of key transcription factors is blocked with the consequent inhibition of cytokine secretion in vitro and sarcomagenesis in vivo [17].

In mice, constitutive activation of Rac-1 in endothelial cells induces cell transformation and development of vascular lesions that are very similar to experimental vascular lesions initiated by constitutive activation of vGPCR. However, these Rac-1 induced vascular lesions do not develop into lesions that microscopically show sheets of spindle cells and vascular slits with extravasated erythrocytes, features characteristic to human KS. This suggests that in addition to constitutive activation of Rac-1, additional vGPCR-mediated signalling pathways are required for the development of KS [17].

By activating the Ras, Raf and Rac intracellular signalling pathways, vGPCR can induce the expression and secretion of VEGF in an autocrine and paracrine manner. This process involves the direct phosphorylation of hypoxia-inducible factor (HIF)-1α, a transcription factor, by both the p38 and MAPK signalling pathways. These pathways are associated with VEGF production, and are present in higher levels in HHV-8 transformed endothelial cells (Diagram 1).

Cytokines and chemokines including IL-1, TNF-α and IL-8 are also able to stimulate the p38 pathway leading to an increased expression of VEGF and bFGF (Diagram 1, red arrows). The increased VEGF expression is sufficient for cell transformation when ectopically expressed in murine fibroblasts. Specific p38 and MAPK inhibitors have been shown to diminish vGPCR-induced VEGF expression and secretion. In addition VEGF is able to rescue cells from apoptosis induced by serum starvation through the activation of the Akt/PKB pathway (Diagram 1, green arrow) [12,15,21,22].

HHV-8 interferon regulatory factor (IRF) inhibits the interferon signalling pathway and is able to induce tumour formation. The expression of p21, a cyclin dependant kinase (CDK) inhibitor, is downregulated by vIRF, which enables the latent virus to evade the immune mediated IFN cell-cycle shutdown, allowing HHV-8 to establish persistence. However, vIRF is not expressed as much in KS as in the cells of Castleman's disease and primary effusion lymphoma, and its role in the development of KS is not clear [29].

It is probable that other viral lytic oncogenes, such as vIL-6, also play a role in HHV-8 mediated cell cycle dysregulation; however, its role in the sarcomagenesis of KS has not yet been defined [29].

#### The clonal nature of HIV-KS lesions

### Human x-linked androgen receptor (HUMARA) gene

Analysis of the inactivation pattern of HUMARA has been used to determine the clonality of KS lesional cells in females [30-33]. This method has three main drawbacks. Firstly, it can only be performed on females, and since KS affects males with a higher frequency than females, this method cannot be used in all cases of KS. Secondly, at times, there is contamination of KS biopsy specimens by infiltration of normal tissue cells. Thirdly, the control samples that the KS samples are compared to, may prove to have unequal x-chromosome inactivation patterns that lead to inaccurate results [31,34].

While some studies using the HUMARA technique showed that the majority of KS cells were monoclonal [32,33], others found that all KS lesions were polyclonal [30] and still others found that KS lesions showed both monoclonal and polyclonal characteristics [31] Therefore, one needs to interpret studies of KS clonality using the HUMARA technique with caution.

### HHV-8 episomal studies

Judde and co-workers [34] studied the clonal nature of HHV-8 terminal repeat sequences in advanced KS lesions and found that such lesions display all patterns of clonality (Table 2). This finding supports the concept that KS starts as a benign angioproliferative disorder comprising a polyclonal cell population, and in later stages of HIV-KS disease, some, if not all KS lesional cells will evolve to a clonal cell population that will undergo expansion [34].

Duprez et al., [35] using the same HHV-8 episomal analysis like Judde et al., [34] demonstrated that most of the advanced KS lesions were oligoclonal (82%), each individual lesion having concurrently multiple HHV-8-infected clones; and some of the advanced KS lesions were monoclonal (18%), each lesion composed of a monoclonal expansion of a single HHV-8 infected cell (Table 3). In addition, individual KS lesions that occur concurrently at different anatomical sites in a subject with multicentric KS, were either monoclonal or oligoclonal; and the size of the HHV-8 episomes varied even between the multicentric monoclonal KS lesions, clearly indicating that these lesions arose from independent clones and were not metastatic lesions originating from the clonal expansion of one HHV-8 infected cell.

**Table 2:** HHV-8 lytic genes

Genes	Products
ORF 74	vGPCR
ORF K6	vMIP-II
ORF K1	vK1
ORF K9	vIRF-1
ORF K8.1	gB

**Table 3:** Results for studies of KS clonality by TR analysis

	**Paper**	**KS cases**	**biopsies**		**Result**		
				monoclonal	oligoclonal	polyclonal	excluded
1)	Judde et al., 2000	26	n = 26	2 (7.6%)	4 (15.3%)	2 (7.6%)	18 (69.2%)

2)	Duprez et al., 2007	98	n = 139	11 (7.9%)	48 (34.5%)	3 (2.1%)	77 (55.3%)

	**Total**	124	n = 165	13 (7.8%)	52 (31.5%)	5 (3%)	95 (57.5%)

Thus KS lesions may be regarded as a reactive proliferation of oligoclonal or monoclonal endothelial cells and not as a true malignancy. The fact that without treatment most subjects with advanced HIV-KS and endemic KS die as a consequence of their KS disease, may be explained by the local aggressive nature of the multicentric lesions. KS lesional cells show uncontrolled proliferation and increased survival. When the lesions affect vital organs such as the heart and the lungs, the disease is fatal [11].

However, one cannot exclude the possibility that in advanced stages of HIV-KS disease, in the presence of other as yet unidentified cofactors cooperating with HHV-8 oncogenes, a subset of the benign monoclonal cells evolve into a malignant clone that undergoes expansion [7].

## Conclusion

From the available data in the literature, one can conclude that HIV-KS starts as a reactive polyclonal angioproliferative response towards HHV-8, and with time, some of the polyclonal cells evolve to form oligoclonal cell populations that undergo expansion. This process is driven by HHV-8 induced intracellular signalling pathways that modulate the expression of cellular genes associated with cell cycle regulation, apoptosis, inflammatory responses and angiogenesis. Whether these benign, reactive, multiple monoclonal cell populations, under certain circumstances, undergo malignant transformation remains to be elucidated.

**Figure 1:**
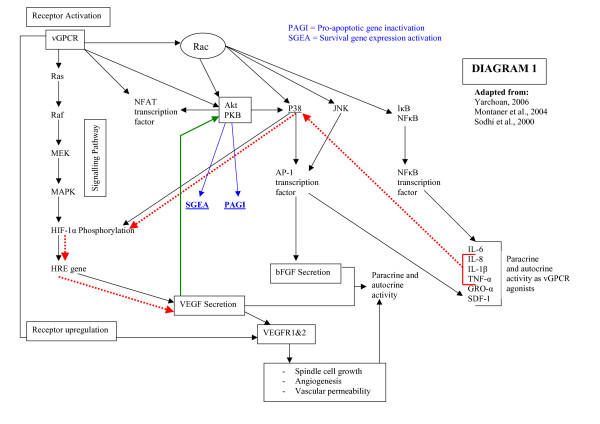
**The signalling pathways mediated by vGPCR**. Constitutive active vGPCR promotes the transcription of inflammatory cytokines and cell transformation. vGPCR through the Rac-NF-κB pathway promotes production of proinflammatory cytokines that in turn leads to recruitment and transformation of neighbouring cells by a paracrine mechanism. The pathway indicated by the red arrows show the activation of the P38 signalling pathway with the consequent increase in VEGF and bFGF production and secretion. The pathway indicated by the blue arrows shows the inactivation of pro-apoptotic genes (PAGI) and the activation of survival genes (SGEA). The green arrow shows activation of Akt/PKB by vGPCR-induced VEGF leading to cell survival.
